# The Role of Drains in Complicated Appendicectomy in Adults: A Narrative Review

**DOI:** 10.7759/cureus.100923

**Published:** 2026-01-06

**Authors:** Narayan Khanal

**Affiliations:** 1 General Surgery, Albury Wodonga Health, Albury, AUS

**Keywords:** abdominal drainage, appendicectomy, complicated appendicitis, intra-abdominal abscesses, surgical drains

## Abstract

Drain placement after appendicectomy for complicated appendicitis remains a subject of ongoing debate. Although drains were traditionally used to prevent intra-abdominal abscesses, more recent evidence indicates limited benefit and potential harm. Substantial international variation persists, and there is no clear consensus regarding indications and clinical benefit. In light of this, we conducted a narrative review of the literature from 2010 to 2025 using MEDLINE, Embase, and the Cochrane Library. Randomised controlled trials, observational studies, systematic reviews, and guidelines evaluating drain use after appendicectomy in complicated appendicitis were included. Evidence was synthesised regarding postoperative intra-abdominal abscess (IAA), wound complications, length of stay, and need for reintervention. Most contemporary studies demonstrate that routine drainage does not reduce postoperative IAA and is associated with increased wound complications, pain, and postoperative hospital length of stay (LOS). Major guidelines generally recommend against routine drainage, favouring irrigation and adequate source control instead. Routine drain placement after perforated appendicitis is not supported by current evidence. Selective use may be considered in limited situations where optimal source control is uncertain. Further high-quality randomised controlled trials are needed to clarify indications and identify subgroups that may benefit.

## Introduction and background

Appendicectomy for acute appendicitis is one of the most common emergency general surgical procedures [1]. Although most cases involve simple inflammation, approximately 30% of patients present with complicated disease, characterised by local perforation, abscess, diffuse peritonitis, or significant contamination [2]. These cases carry a higher risk of postoperative intra-abdominal abscess (IAA), surgical site infections (SSI), prolonged hospital length of stay (LOS), and mortality [3].

Historically, the use of intra-abdominal drains after contaminated abdominal surgery was based on the belief that passive or active drainage would evacuate residual infected fluid, reduce bacterial load, and prevent abscess formation [4]. This rationale predated the widespread use of cross-sectional imaging, modern antibiotics, and minimally invasive surgery, when postoperative intra-abdominal sepsis was often diagnosed late and associated with high mortality [5,6]. As a result, drain placement became ingrained in surgical training and practice, particularly in cases of perforation or diffuse peritonitis. However, the role of drains has been increasingly questioned [7]. Advances in minimally invasive techniques, improved source control, postoperative antibiotics, and interventional radiology have significantly changed contemporary management strategies [8,9].

Recent studies suggest that drains may not provide benefit and may instead increase postoperative pain, wound complications, and length of stay [10,11]. Despite evolving evidence, drain use remains highly variable across institutions and surgeons. Some continue to place drains selectively in high-risk situations such as gross contamination, friable tissue, or inadequate source control, whereas others have abandoned routine drainage entirely [12,13]. This variation reflects ongoing uncertainty regarding indications, duration, and clinical benefit of drains following appendicectomy. This narrative review synthesises evidence regarding drain use after appendicectomy for complicated appendicitis. It evaluates comparative outcomes, examines international guideline recommendations, and identifies remaining gaps in knowledge.

## Review

Methods

Study Design

A narrative review was undertaken to synthesise evidence from randomised trials, observational studies, meta-analyses, and guideline statements evaluating surgical drain use after appendicectomy.

The review was designed to address three key clinical questions: 1. Does drain placement reduce postoperative intra-abdominal abscess in complicated appendicitis? 2. What are the effects of drains on wound infection, postoperative pain, LOS, and reintervention? 3. How consistent are guideline recommendations regarding drainage?

Search Strategy

A structured literature search was performed across the following electronic databases: MEDLINE (via PubMed), Embase, andCochrane Library. The search included studies published between January 2010 and December 2025. Earlier landmark studies were included where appropriate to provide historical context or where data remain relevant to contemporary practice. The following search terms and Boolean combinations were applied: “appendicectomy” OR “appendicectomy” AND “drain”; “perforated appendicitis” AND “intra-abdominal abscess”; “complicated appendicitis” AND “postoperative complications”; “surgical drains” AND “general surgery”; and “laparoscopic appendicectomy” AND “drain placement”.

Reference lists of relevant systematic reviews and guidelines were manually screened to identify additional publications not captured by initial database searches. Search syntax was adapted for each database using combinations of controlled vocabulary (e.g., Medical Subject Headings (MeSH) terms) and free-text keywords, with Boolean operators (“AND”, “OR”) applied according to database-specific conventions.

Inclusion Criteria

Studies were included if they met all of the following criteria: studies evaluating drain placement after complicated appendicectomy; RCTs, cohorts, case-control studies, systematic reviews, and meta-analyses; and studies reporting key outcomes: IAA, SSI, LOS, and reintervention

Exclusion Criteria

The exclusion criteria were as follows: non-English papers; case series involving <20 patients; studies focusing solely on simple appendicitis; paediatric-only studies; and animal studies

Definition of Complicated Appendicitis

For this review, “complicated appendicitis” was defined as patients with at least one of the following: perforated appendix, purulent or faeculent contamination, localised or generalised peritonitis, gangrenous appendicitis, intraoperative abscess, appendicular mass

This classification reflects definitions used across multiple major trials and guideline frameworks. To assess practice variation, international guidelines were reviewed. No formal risk-of-bias assessment tool was applied to individual studies, as this review employed a narrative synthesis of heterogeneous study designs rather than a quantitative systematic approach. This is acknowledged as a limitation of the review methodology.

Ethical Considerations

Ethics approval was not required as this review utilised publicly available data and did not involve patient-identifiable information or institutional data collection.

Evidence

Impact on Postoperative Intra-abdominal Abscess (IAA)

Across the literature, the most consistently evaluated outcome is postoperative IAA. Multiple randomised and observational studies indicate that routine drainage does not reduce IAA rates following complicated appendicectomy. Foreign-body colonisation, persistent leakage, and tract contamination have been proposed as mechanisms [14]. A 2022 systematic review and meta-analysis of 17 studies and over 4,000 patients by Abu et al. demonstrated comparable IAA rates between drainage and non-drainage cohorts, with several included studies showing numerically higher abscess rates in the drainage group. Importantly, this analysis incorporated both laparoscopic and open approaches, suggesting that the lack of benefit persists across operative techniques [14].

Similarly, post hoc analysis of a large multicentre EAST dataset by Qian et al. found no protective effect of drainage against abscess formation, even after adjusting for disease severity and contamination, reinforcing the conclusion that drains do not mitigate the principal complication they are intended to prevent [15]. More recent laparoscopic-specific studies further challenge the rationale for routine drainage. Liao et al. demonstrated a significantly higher rate of postoperative IAA and prolonged hospital stay among patients receiving drains following laparoscopic appendicectomy for complicated appendicitis in this observational cohort [16]. These findings are particularly relevant to modern practice, as laparoscopic appendicectomy now constitutes the predominant approach in most health systems. Collectively, these data indicate that routine drainage not only fails to reduce IAA but may paradoxically be associated with worse intra-abdominal infectious outcomes in contemporary cohorts.

Effect on SSI, Pain, LOS, and Readmission

Beyond IAA, a growing body of evidence demonstrates that drain placement is associated with increased postoperative morbidity. Multiple studies report higher wound-related complications in drained patients. Drains may act as bacterial entry points, particularly with open or prolonged operations. A meta-analysis by Abu et al. identified a significant increase in SSI, fistula, bowel obstruction, ileus, and hospital LOS when drains were used, highlighting that the harms of drainage extend beyond minor discomfort [14]. Several studies, including systematic reviews and meta-analyses, have consistently demonstrated that hospital LOS is prolonged in patients receiving drains, with pooled analyses reporting increases ranging from approximately one to three additional inpatient days [13,17,18,19,20].

This prolongation is clinically meaningful in the context of modern surgical care, where early mobilisation and enhanced recovery pathways are prioritised. Contributing factors for increased LOS include prolonged antibiotic use, pain from the drain, and delayed mobilisation [20]. Furthermore, pain scores also appear worse in drained patients. Groothoff et al. found that pain scores are higher in the drainage group compared to the non-drainage group [21]. A common argument in favour of drains is the perceived reduction in need for delayed reintervention [18]. However, available evidence does not support this assumption. Large observational studies demonstrate no reduction in readmission rates, percutaneous drainage, or reoperation among patients with prophylactic drains [15].

The impact of drain placement must also be interpreted in the context of the operative approach. Many earlier studies supporting drainage were conducted in the open appendicectomy era, when visualisation of the peritoneal cavity was limited. In contrast, laparoscopic appendicectomy offers superior visualisation, targeted suction and thorough irrigation, reducing residual contamination [22]. Contemporary studies conducted predominantly in the laparoscopic era consistently demonstrate no benefit of routine drainage and suggest increased postoperative pain and LOS in drained patients, indicating that conclusions drawn from open-era studies may not apply to modern practice. Table 1 presents a summary of studies assessing the use of drains in complicated appendicectomy.

**Table 1:** Summary of studies assessing the use of drains in complicated appendicectomy LOS: length of stay; OR: odds ratio; SSI: surgical site infection; CI: confidence interval; MD: mean difference; length of hospitalisation; SMD: standardised mean difference; WI: wound infection; PI: postoperative ileus; RR: risk ratio

Author	Year	Study type	Sample	Key findings
Nazarian et al. [13]	2021	Retrospective review	N = 76	Postoperative complication: 9 (34.6%) vs. 6 (12%), p = 0.019; intra-abdominal abscess: 5 (19.2%) vs. 3 (6%), p = 0.07; and LOS: 5.5 days vs. 3 days, p = 0.0001 were significantly higher in patients with a drain
Abu et al. [14]	2018	Systematic review and meta-analysis	N = 4,255	There was no significant difference between the two groups regarding abdominal collection (OR = 1.41, p = 0.13). No-drain group was superior to the drain group regarding SSI (OR = 1.93, p = 0.0001), faecal fistula (OR = 4.76, p = 0.03), intestinal obstruction (OR = 2.40, p = 0.04), and paralytic ileus (OR = 2.07, p = 0.01)
Qian et al. [15]	2021	Prospective study	N = 634	Drain group had a higher complication rate (43% vs. 28%; p = 0.001) and longer LOS (4 [3–7] vs. 3 [1–5] days
Liao et al. [16]	2022	Retrospective cohort study	N = 1,241	For complicated appendicitis, the drainage group (n = 192) tended to harbor more overall complications, intra-abdominal abscess formation, time to resume a soft diet, and the postoperative length of hospitalisation (p = 0.0000 for all). Multivariate logistic regression confirmed that abdominal drainage increased the risk of overall complications (OR = 2.439; 95% CI = 1.597–3.726; p ≤ 0.0001) and failed to decrease the risk of intra-abdominal abscess formation (OR = 1.655; 95% CI = 0.487–5.616; p = 0.4193)
Cheng et al. [17]	2015	Cochrane review	N = 453	There were no significant differences between the two groups in the rates of intra‐peritoneal abscess or wound infection. The hospital stay was longer in the drainage group than in the no drainage group (MD = 2.04 days; 95% CI = 1.46–2.62) (34.4% increase of an 'average' hospital stay)
Liao et al. [18]	2023	Meta analysis	N = 5,123	Compared with patients in the non-drainage group, patients in the drainage group had longer postoperative LOH (SMD = 0.68, 95% CI = 0.01–1.35, p = 0.046), higher overall incidence of postoperative complications (OR = 0.50, 95% CI = 0.19–0.81, p = 0.01), higher incidence of WI (OR = 0.30, 95% CI = 0.08–0.51, p = 0.01) and PI (OR = 1.05, 95% CI = 0.57–1.54, p = 0.01); the differences were statistically significant
Tang et al. [19]	2025	Cochrane review	N = 739	The evidence is very uncertain regarding the effects of abdominal drainage versus no drainage on intraperitoneal abscess at 30 days (RR = 1.08, 95% CI = 0.55 to 2.12; seven studies, 671 participants; very low‐certainty evidence), wound infection at 30 days (RR = 1.76, 95% CI = 0.89 to 3.45; seven studies, 696 participants), and morbidity at 30 days (RR = 1.84, 95% CI = 0.14 to 24.50; two studies, 124 participants). Approximately 113 (57 to 221 participants) out of 1,000 participants in the drainage group developed an intraperitoneal abscess, compared with 104 out of 1,000 participants in the no‐drainage group. There were seven deaths in the drainage group (n = 291) compared with one in the no‐drainage group (n = 290); abdominal drainage probably increases the risk of 30‐day mortality (Peto odds ratio = 4.88, 95% CI = 1.18–20.09; six studies, 581 participants; moderate‐certainty evidence)
Lu et al. [20]	2025	Retrospective analysis	N = 128	The abdominal drain group exhibited significantly longer operative time (p = 0.010), duration of postoperative antibiotic use (p < 0.001), and LOS (p < 0.001)

Guidelines Recommendations

Major international guideline bodies have shifted away from recommending routine drain placement following appendicectomy for complicated appendicitis, reflecting an evolving evidence base. The European Association for Endoscopic Surgery (EAES), in its 2015 consensus on the management of complicated appendicitis, advises that drain use is not advocated and recommends primary attention to adequate source control [23]. Similarly, the World Society of Emergency Surgery (WSES), in the 2020 update of its “Jerusalem guidelines”, explicitly recommends against routine abdominal drains for complicated appendicitis or abscess or peritonitis in adults; however, it emphasises surgeon discretion in selected cases. The guideline notes that available randomised and observational data fail to demonstrate any benefit from drains [24].

Discussion

This narrative review synthesised contemporary evidence on drain placement after complicated appendicectomy. The overall conclusion based on systematic reviews, meta-analyses, large cohorts, and laparoscopic-era studies is consistent: intra-abdominal drainage does not reduce postoperative IAA and is associated with harms, including higher rates of SSI, greater pain, and longer LOS in hospital. These findings underpin recent guideline recommendations that discourage drain use [14,15,18,19,23,24]. The consistency of these findings across study designs and clinical settings strengthens the inference that routine drainage offers no clinically meaningful benefit. Notably, the absence of IAA reduction has been demonstrated in randomised trials, propensity score-matched analyses, and large multicentre cohorts, including studies that adjusted for disease severity and degree of contamination. This reduces the likelihood that the observed lack of benefit is attributable solely to confounding by indication. Furthermore, several studies report numerically higher abscess rates in drained patients, suggesting that drainage may be associated with worse outcomes in selected contexts.

Several pathophysiological mechanisms may explain the lack of benefit observed with routine drain placement. Within a short period following placement, drains may become obstructed by fibrin, blood, pus, debris, and clots [10]. Moreover, the presence of a foreign body within the peritoneal cavity may paradoxically increase infection risk rather than mitigating it [25]. Drains may also act as a conduit for retrograde contamination from the skin into the peritoneal cavity, particularly when left in situ for prolonged periods [26]. These mechanisms align with clinical findings demonstrating no reduction in IAA and, in some cases, higher complication rates in drained patients.

A plausible explanation for the lack of benefit is that modern management of complicated appendicitis prioritises effective source control, including complete removal of necrotic tissue and suction of pus, appropriate perioperative antibiotics, and the early use of image-guided drainage when abscesses develop [27]. Advances in laparoscopic techniques have significantly improved visualisation of the peritoneal cavity, allowing more targeted suction of dependent collections and a more thorough irrigation compared with historical open approaches. When these elements are optimised, an intraoperative drain adds little in reducing collection formation but does add a foreign body that may act as a conduit for bacteria and increase patient discomfort and immobilisation, with these mechanisms supported by pooled analyses and cohort data [14,16,17].

Therefore, a practical, evidence-informed approach is to adopt a selective drainage strategy and reserve drains for uncommon situations in which adequate source control is not achievable intraoperatively, such as an inability to clear frank faecal contamination or when a large, irregular cavity cannot be adequately evacuated or approximated, with clear documentation of the rationale and planned duration. If drains are used, they should be removed early when output is low and clinical progress is satisfactory, as evidence demonstrates that prolonged drain placement carries a significant risk of complications [14,17,20,28]. This approach balances clinician concerns about delayed need for reintervention with the evidence of drain-related harms.

Limitations

The literature has several important limitations that weaken the strength of conclusions. Much of the comparative data are observational and subject to selection bias, as surgeons preferentially insert drains in sicker patients, which can exaggerate harms in the drained cohort. Definitions of “complicated” disease, drain type and duration, outcome measures, follow-up intervals, and perioperative antibiotic regimens vary widely between studies, particularly regarding gangrenous appendicitis without overt perforation. While some classification systems consider non-perforated gangrenous appendicitis as distinct from perforated disease, others include it within the spectrum of complicated appendicitis due to its association with higher postoperative complications [14,16,29,30,31]. Randomised controlled trial data are limited in number and often small, and many datasets combine open and laparoscopic approaches, complicating direct applicability to specific patient groups.

Despite the overall trend against routine drainage, uncertainty persists for specific subgroups. For example, patients with massive faecal contamination, extensive necrosis at the appendiceal base, or those requiring conversion to open surgery were under-represented in high-quality trials and may potentially benefit from selective drainage, although robust evidence is lacking [16,21]. Likewise, resource-limited settings where access to timely image-guided drainage is restricted might reasonably adopt a different risk threshold. These nuances highlight why existing surveys show ongoing practice variation, with clinician experience, local resources, and perceived patient risk continuing to drive decision-making [20].

Recommendations

To address remaining uncertainties, future research should prioritise a pragmatic, tiered approach. While well-powered, multicentre randomised controlled trials remain appropriate for selected patients with complicated appendicitis where genuine clinical equipoise exists, their feasibility in the most severe subgroups - such as faecal peritonitis, extensive gangrene, or gross contamination - is limited by ethical and recruitment challenges. In these high-risk contexts, large-scale prospective registry studies and standardised data collection initiatives are likely to provide more feasible and generalisable evidence. Such registries should capture granular intraoperative findings, disease severity, rationale for drain placement, drain type and duration, perioperative antibiotic strategies, and patient-centred outcomes, including pain, time to mobilisation, and quality of life. In parallel, economic evaluations comparing routine drain, selective drain, and no-drain strategies should be incorporated to account for downstream costs related to prolonged hospitalisation, radiological interventions, and hospital readmissions. Finally, further investigation into optimal postoperative antibiotic strategies in the presence or absence of drains is warranted to minimise unnecessarily prolonged therapy, reduce antimicrobial resistance, and shorten hospital length of stay.

## Conclusions

Current evidence and international guideline recommendations converge in advising against drain placement after complicated appendicectomy due to the risk of higher SSI, increased pain, and longer hospital LOS. Selective drainage, based on clearly documented intraoperative concerns about source control or anatomy, remains reasonable but should be rare. High-quality trials and prospective registries are needed to define the small subgroup, if any, that may benefit from intraoperative drainage and to refine practical protocols, including drain types, management, and removal timing.
